# CD146^+^ Human Umbilical Cord Perivascular Cells Maintain Stemness under Hypoxia and as a Cell Source for Skeletal Regeneration

**DOI:** 10.1371/journal.pone.0076153

**Published:** 2013-10-18

**Authors:** Wing Pui Tsang, Yinglan Shu, Po Lam Kwok, Fengjie Zhang, Kenneth Ka Ho Lee, Mei Kuen Tang, Gang Li, Kai Ming Chan, Wai-Yee Chan, Chao Wan

**Affiliations:** 1 Key Laboratory for Regenerative Medicine, Ministry of Education, School of Biomedical Sciences, Faculty of Medicine, The Chinese University of Hong Kong, Hong Kong SAR, China; 2 School of Biomedical Sciences Core Laboratory, The Chinese University of Hong Kong Shenzhen Research Institute, Shenzhen, China; 3 Department of Orthopaedics and Traumatology, The Chinese University of Hong Kong, Hong Kong SAR, China; University of Kansas Medical Center, United States of America

## Abstract

The human umbilical cord perivascular cells (HUCPVCs) have been considered as an alternative source of mesenchymal progenitors for cell based regenerative medicine. However, the biological properties of these cells remain to be well characterized. In the present study, HUCPVCs were isolated and sorted by CD146^+^ pericyte marker. The purified CD146^+^ HUCPVCs were induced to differentiate efficiently into osteoblast, chondrocyte and adipocyte lineages *in vitro*. Six weeks following subcutaneous transplantation of CD146^+^ HUCPVCs-Gelfoam-alginate 3D complexes in severe combined immunodeficiency (SCID) mice, newly formed bone matrix with embedded osteocytes of donor origin was observed. The functional engraftment of CD146^+^ HUCPVCs in the new bone regenerates was further confirmed in a critical-sized bone defect model in SCID mice. Hypoxic conditions suppressed osteogenic differentiation while increased cell proliferation and colony-forming efficiency of CD146^+^ HUCPVCs as compared to that under normoxic conditions. Re-oxygenation restored the multi-differentiation potential of the CD146^+^ HUCPVCs. Western blot analysis revealed an upregulation of HIF-1α, HIF-2α, and OCT-4 protein expression in CD146^+^ HUCPVCs under hypoxia, while there was no remarkable change in SOX2 and NANOG expression. The gene expression profiles of stem cell transcription factors between cells treated by normoxia and hypoxic conditions were compared by PCR array analysis. Intriguingly, PPAR-γ was dramatically downregulated (20-fold) in mRNA expression under hypoxia, and was revealed to possess a putative binding site in the Hif-2α gene promoter region. Chromatin immunoprecipitation assays confirmed the binding of PPAR-γ protein to the Hif-2α promoter and the binding was suppressed by hypoxia treatment. Luciferase reporter assay showed that the Hif-2α promoter activity was suppressed by PPAR expression. Thus, PPAR-γ may involve in the regulation of HIF-2α for stemness maintenance and promoting the expansion of CD146^+^ HUCPVCs in response to hypoxia. CD146^+^ HUCPVCs may serve as a potential autologous cell source for bone regeneration.

## Introduction

Adult stem cells represent a promising model for regenerative medicine and tissue engineering. Mesenchymal stem cells (MSCs) derived from adult bone marrow have been considered as the most common cell source for cell-based therapies. MSCs are multipotent stromal cells that can differentiate into variety of mesenchymal lineages, including osteoblasts, chondrocytes and adipocytes [1]–[7]. MSCs based cell therapy has been widely used in musculoskeletal repair [8]–[10], myocardium regeneration [11] and dermal wound healing [12]. However, current sources of MSCs require invasive procurement procedures and provide relatively low frequency of progenitors. To overcome these limitations, human umbilical cord perivascular cells (HUCPVCs) have been considered as an alternative source of mesenchymal progenitors. HUCPVCs have been shown to have a high proliferation potential and colony formation capacity *in vitro*. Moreover, they proliferate and differentiate rapidly into an osteogenic phenotype in culture by addition of osteogenic supplements such as dexamethasone [13]. In fact, HUCPVCs have been shown to undergo multilineage differentiation and contribute to repair of osteochondral defects *in vivo* [14]. Transplanted HUCPVCs not only contribute to tissue healing and matrix generation themselves, but also are able to recruit resident progenitors to rapidly repair the damaged tissues [14]. These evidences indicate that HUCPVCs may function as a potential cell source for tissue regeneration therapeutics.

Stem cells reside in a defined microenvironment which is also known as “stem cell niches”. Stem cell niches play a critical role in stem cell self-renewal and fate determination. Studies on different stem cell niches indicate that the oxygen tension of different tissues or organs in which stem cells exist are ranging from 1% to 8%, which is far below the atmospheric oxygen tension (20%). For instance, the inner cell mass derived embryonic stem cells (ESCs) exist in 3%–5% oxygen tension [15]. The oxygen level in mammalian brain tissue ranges from 1%–5% [16]. It is believed that a relatively hypoxic microenvironment is crucial for the maintenance of cell “stemness”, i.e., sustaining the stem cells in an undifferentiated state for proliferation, differentiation and self-renewal. One of the mechanisms for regulation of stem cell pluripotency in hypoxic conditions involves the upregulation of hypoxia inducible factors (HIFs). It has been shown that HIF-2α but not HIF-1α is a direct upstream regulator of Oct-4, a transcription factor which is essential for maintaining ESCs pluripotency [17], [18]. HIF-2α was reported to bind to the Oct-4 promoter for regulating Oct-4 expression in ESCs [17]. An increase in Oct-4 expression after hypoxia exposure was also observed in mesenchymal stem cells [19]. In addition to Oct-4, Sox2 and Nanog were also found to be regulated by HIF-2α [18]. These transcription factors are pluripotency markers which play an important role in maintaining stemness by repressing genes that promote differentiation [20]. In fact, interactions between hypoxia-responsive transcription factors with other transcription factors in regulation of various cellular responses have been reported. For instance, NF-kB can transactivate HIF-1α directly in inflammatory responses [21]. HIF-1α and AP-1 cooperate to regulate gene expression in hypoxia [22]. However, little is known about the interactions between stem cell transcription factors and hypoxia-responsive genes.

In the present study, CD146^+^ HUCPVCs were purified and enriched. The multilineage differentiation potential of this population was characterized under both normoxic and hypoxic conditions *in vitro*. The osteogenic differentiation and functional engraftment of CD146^+^ HUCPVCs in the new bone regenerates were demonstrated in the *in vivo* bone formation model in SCID mice. Hypoxia was shown to increase the colony forming efficiency and proliferation of CD146^+^ HUCPVCs, and modulate their osteogenic differentiation. Gene expression profiles of stem cell transcription factors between normoxic and hypoxic environment were compared by PCR array to explore the hypoxia-responsive genes in controlling stem cell self-renewal and proliferation. We also provided the evidence of the occupancy and transcriptional suppression of the Hif-2α promoter by PPARγ, indicating that the expansion of CD146^+^ HUCPVCs under hypoxia was mediated by coordinated suppression of PPARγ and upregulation of HIF-2α expression.

## Materials and Methods

### Isolation and purification of HUCPVCs from human umbilical cord

HUCPVCs were isolated from human umbilical cords obtained from the Department of Obstetrics and Gynecology, Prince of Wales Hospital of The Chinese University of Hong Kong and were approved by The Chinese University of Hong Kong Clinical Research Ethics committee. This is centrally registered with Hong Kong Health Authority. The physician obtained verbal informed consent from the mother for use of the umbilical cord in medical research. Here we clarify that the verbal informed consent and the procedure were approved by The Chinese University of Hong Kong Clinical Research Ethics committee (project reference number CRE 2011.116). The umbilical cords were carefully cut open and the blood vessels were dissected. Sutures were made at both ends of the vessels and the tied vessels were put in a 15 ml centrifuge tube containing a solution of 1 mg/ml of collagenase (Sigma) in PBS. After 16 hours, the vessels were removed and the tube was centrifuged at 400× g for 5 minutes to collect the digested cells for further purification. HUCPVCs were purified with CD146 antibody from the heterogenous primary culture at passage two using the Dynal CD146 Progenitor Cell Selection System (Invitrogen). Briefly, HUCPVCs were resuspended in isolation buffer (100 mM PBS, 0.1% BSA and 2 mM EDTA, pH 7.4) at a concentration of 1×10^8^ cells/ml. CD146 antibody-coated magnetic beads was added to the cell suspension and was incubated at 4°C for 30 minutes with gentle rotation. The mixture tube was then placed on a magnetic stand (Invitrogen) for attraction of bead-bound cell population. The purified CD146^+^ HUCPVCs were then collected by centrifugation. Cells were resuspended and maintained in DMEM supplemented with 15% embryonic stem cells qualified-fetal bovine serum (ESQ-FBS) (Invitrogen) and 0.01 mg/ml penicillin-streptomycin.

### Immunophenotyping

Cells grown on 12-mm coverslips were washed twice with PBS and fixed with 10% formalin. Cells were permeablized by incubating with 2 M HCl with 0.5% (v/v) Triton X-100 for 30 min and pre-blocked with 2% BSA for 1 hr. Immunofluorescence staining was performed by an incubation with rabbit anti-CD146 (Zymed) antibody overnight at 4°C, followed by Alexa Fluor 647 secondary antibody (Invitrogen) for 1 hr. Cells were washed extensively with 0.1% tween-20 in PBS and mounted with DAPI (Molecular Probes) nuclear stain in 50% (v/v) glycerol. Fluorescent signal was visualized by Olympus FV1000 confocal microscope (Olympus). The image was processed by using FV10-ASW software (Olympus). The cell surface epitope profile (CD44, CD90, CD105, CD146, CD34 and CD45) of CD146^+^ HUCPVCs were examined by flow cytometric analysis. Briefly, the cells were trypsinized, washed twice with PBS and then incubated with conjugated mouse monoclonal antibodies to human CD44-PE, CD90-PE (Thy-1), CD146-PE, CD34-PE, CD45-PE, or unconjugated antibodies to human CD105 (SH2) (all from BD Biosciences) for 30 min at 4°C. After washing with 2% FBS/PBS, cells stained with CD105 were incubated with anti-mouse FITC-conjugated secondary antibody (BD Bioscience) for 20 min at 4°C. The cells were washed twice and then resuspended in 2% FBS/PBS for flow cytometry analysis (LSRFortessa Analyzer, BD Bioscience). Data were analyzed with FACS Diva software.

### Quantitative real-time polymerase chain reaction (qPCR)

Total RNA was extracted using TRIzol reagent (Invitrogen). First strand cDNA was synthesized from 1 µg of total RNA in the presence of oligo-dT_12–18_ primer (Invitrogen) and MMLV reverse transcriptase according to manufacturer's instructions (Promega). Quantitative real-time PCR was performed with SYBR Premix Ex Taq (Takara) in ABI Fast Real-time PCR 7900HT System (Applied Biosystems). All samples were performed in triplicates. β-actin was amplified in parallel as an endogenous control. Primer sequences were shown in Table 1.

**Table 1:** Sequences of primers used for quantitative real-time PCR.

Gene		DNA sequence (5′ to 3′)
Cebpα	Forward	ACTGGGACCCTCAGCCTTG
	Reverse	TGGACTGATCGTGCTTCGTG
Ppar- γ	Forward	CAGGAAAGACAACAGACAAATCA
	Reverse	GGGGTGATGTGTTTGAACTTG
Fabp4	Forward	CATCAGTGTGAATGGGGATG
	Reverse	ATGCGAACTTCAGTCCAGGT
Sox-9	Forward	GCCAGGTGCTCAAAGGCTA
	Reverse	TCTCGTTCAGAAGTCTCCAGAG
Col2α1	Forward	ACACTGGGACTGTCCTCTG
	Reverse	GTCCAGGGGCACCTTTTTCA
Col10α1	Forward	ATGCTGCCACAAATACCCTTT
	Reverse	GGAATGAAGAACTGTGTCTTGGT
Alp	Forward	CACCCACGTCGATTGCATCT
	Reverse	TAGCCACGTTGGTGTTGAGC
Runx2	Forward	GCCTAGGCGCATTTCAGA
	Reverse	CTGAGAGTGGAAGGCCAGAG
Ocn	Forward	GGCGCTACCTGTATCAATGG
	Reverse	TCAGCCAACTCGTCACAGTC
Col1α1	Forward	GAGAGCATGACCGATGGATT
	Reverse	ATGTAGGCCACGCTGTTCTT
Osx	Forward	ATGTCTTGCCCCAAGATGTC
	Reverse	TATCCACCACTACCCCCAGT
β-actin	Forward	GTTGTCGACGACGAGCG
	Reverse	GCACAGAGCCTCGCCTT

### *In vitro* multilineage differentiation

CD146^+^ HUCPVCs were seeded in 6-well culture plates and cultured until confluency. Osteogenic differentiation was induced by culturing the cells for 7 and 14 days in complete medium with 1 nM dexamethasone, 50 mM ascorbic acid and 20 mM β-glycerophosphate. Adipocyte differentiation was induced with 100 nM dexamethasone, 0.5 mM methyl-isobutylxanthine (IBMX), 50 µM indomethacin and 10 µg/ml insulin (all from Sigma-Aldrich) for 7, 14 and 28 days respectively. Chondrogenic differentiation was induced with 1 µM dexamethasone, 0.2 mM ascorbic acid, 1 mM sodium pyruvate (all from Sigma-Aldrich), ITS^+^ Premix in 1∶100 dilution (BD Biosciences) and 10 ng/ml TGF-β (eBioscience) for 7, 14 and 28 days respectively. All cultures were maintained with 5% CO2 at 37°C. Medium changes was performed twice weekly. In a parallel study, CD146^+^ HUCPVCs were cultured under hypoxia (2% oxygen) for two passages, with 7 days for each passage. Expanded CD146^+^ HUCPVCs under hypoxia were further processed for multilineage differentiation analysis using the above mentioned conditions under normoxic conditions for up to 14 days. For further analysis, expanded CD146^+^ HUCPVCs from each patient were divided into two groups, one group was cultured under normoxia and the other group simultaneously was cultured under hypoxia.

### Cytochemical staining

Cells were washed with PBS and fixed with 10% formalin for 30 min. For detection of alkaline phosphatase activity, cells were stained with NBT-BCIP (Pierce) for at least 10 min or until desire color was developed. The reaction was stopped by washing the cells with distilled water. Mineralization of osteoblastic lineage was detected by staining with 1% Alizarin Red S (Sigma-Aldrich) for 30 minutes. The presence of proteoglycans during chondrogenic differentiation was detected by staining with 3% Alcian blue (Sigma-Aldrich) for 1 hr. Adipocytes were stained with Oil Red O as follows: cells were washed with 60% isopropanol for 5 minutes after fixed with 10% formalin as described. Thereafter cells were stained in Oil Red O (Sigma-Aldrich) working solution (3 portions of 0.5% Oil Red O in isopropanol to 2 portions of distilled water) for 15 minutes. All staining procedures were performed at room temperature. After staining cells were washed extensively with distilled water.

### Scanning electron microscopy (SEM)

Cells cultured on 25-mm coverslips were washed with PBS and fixed with 2.5% glutaraldehyde for 4 hr at room temperature. Cells were then post-fixed with 1% osmium tetraoxide for 15 min, dehydrated by a graded series of ethanol and subjected to critical point drying with liquid carbon dioxide. After coating with palladium-gold, the samples were examined under a JSM-6301F scanning electron microscope (Joel).

### Colony formation assay

500 cells were plated in 25 cm^2^ culture dishes for 24 h and then cultured in a routine water-jet CO_2_ incubator or hypoxic (2% oxygen) incubator (New Brunswick) supplied with 5% CO_2_ for 2 weeks. Cells were washed with PBS, fixed with 10% formalin for 20 min and stained with 0.5% crystal violet (Sigma) for 1 h. The whole image was scanned and total number of colonies in each treatment was counted. Cell morphologies were captured by phase contrast microscopy.

### BrdU incorporation assay

Cell proliferation was examined by using BrdU incorporation assay according to the manufacturer's instructions. Briefly, the cells were washed, fixed and labeled with BrdU for 4 h. Thereafter the cells were incubated with peroxidase-conjugated anti-BrdU antibody for 90 min. BrdU incorporation was detected by incubating the cells with tetramethyl-benzidine (TMB) as a substrate. Color development, which was directly proportional to the amount of DNA synthesis and hereby to the number of proliferating cells, was quantified by measuring the absorbance at 370 nm by a microplate reader.

### PCR array analysis

Quantitative PCR array analysis was performed using mouse Stem Cell Transcription Factors RT2 Profiler PCR Array to examine the expression of 84 genes (Qiagen). Cells were cultured in normoxia (20% oxygen) or hypoxia (2% oxygen) for 24 h. Total RNA was extracted, reverse transcribed and PCR amplified according to manufacturer's instructions. Hierarchical cluster analysis was performed by MeV 4.8.1 software using neighbor-joining method with Pearson correlation coefficient to measure the similarity between gene profiles.

### Western blot

Cells were washed three times with ice-old PBS, lyzed in Lammeli's lysis buffer containing 1% Triton X-100, and scraped by a cell lifter. Protein concentration was examined by BCA protein assay (Pierce). 50 µg of total protein was denatured, resolved in 15% SDS-PAGE minigel, and electrophoretically transferred onto an Immobilon-P membrane (Millipore). The membrane was blocked with 5% nonfat dry milk and hybridized with antibodies against PPAR-γ (Abcam), HIF-1α (Novus), HIF-2α (Novus), SOX2 (Millipore), NANOG (Abcam) or OCT4 (Santa Cruz) at 4°C overnight. Membranes were washed extensively with 0.1% Tween-20 in PBS, and then incubated with horse-radish peroxidase conjugated secondary antibody (Molecular Probes) in 1∶10000 dilution at room temperature for 1 h. Protein expression signals were detected with enhanced chemiluminescence (GE Healthcare) and developed on an x-ray film (Roche).

### Chromatin immunoprecipitation (ChIP)

ChIP assay was performed using ChIP assay kit (Upstate) according to the manufacturer's instructions. 2×10^6^ cells were cross-linked with 1% formaldehyde, washed with PBS and lysed in SDS lysis buffer with protease inhibitors. The lysates were sonicated to shear chromosomal DNA into less than 500 bp. The diluted chromatin was precleared by incubating with protein A agarose/salmon sperm DNA for 30 min at 4°C with agitation. Immunoprecipitation was performed by binding 5 µg of mouse anti-PPARγ antibody (Abcam) to the lysates and incubated at 4°C overnight with rotation. Immune complexes were pulled down by agitating with protein A agarose/salmon sperm DNA for 1 h, washed and then eluted in the buffer containing 1% SDS. Chromatin DNA was de-crosslinked and purified by phenol/chloroform extraction. Purified DNA was dissolved in 40 µl TE buffer. 1 µl was used for quantitative PCR reaction to detect the presence of Hif-2α promoter. DNA sequence for the primer pair is (Forward) 5′-TCTGGTACCTGCATGCCATA-3′ and (Reverse) 5′-GTCATTGTTCCTGGCTGACC-3′ respectively. The percentage of DNA bound was calculated with the total amount of DNA purified from the chromatin (input DNA).

### *In vivo* bone formation model

Experimental procedures were carried out by protocols approved by Animal Experimentation Ethics Committee and Animal (Control of Experiments) Ordinance from Department of Health, Hong Kong SAR. CD146^+^ HUCPVCs (2×10^6^ cells) were suspended in 4% alginate acid sodium (Sigma) solution supplemented with 1 µg recombinant human bone morphogenetic protein 2 (rhBMP2) and carefully added into 3D Gelfoam scaffold (3×3×3 mm). Then the Gelfoam was added in 5 mM calcium chloride (Sigma) solution to generate the 3D CD146^+^ HUCPVCs-Gelfoam-alginate complexes. The implants were then incubated in 37°C for 30 min and transplanted into a subcutaneous pocket created by a 5 mm-long skin incision on the back of the SCID mice. The wounds were carefully closed with standard surgical procedure. After 6 weeks of recovery, the mice were sacrificed and the samples were collected for histological analysis. Radiographs were performed by using a Faxitron X-ray machine.

A critical sized bone defect model was performed in the SCID mice using standard surgical procedures. Briefly, the SCID mice were anesthetized and the right femurs of the mice were fixed with a fixator and a 3.0 mm-long segmental defect was created in each mouse. The CD146^+^ HUCPVCs-Gelfoam-alginate 3D complexes were transplanted into the bone defects. The wounds were closed using standard surgical procedure. After 6 weeks, the mice were euthanized and the transplants were harvested for radiographic analysis using a Faxitron X-ray machine, and then processed for histological analysis.

### Histology and immunohistochemistry

The subcutaneous explants or whole femurs with bone defect were harvested, fixed in 10% buffered formalin and decalcified in 10% EDTA at pH 7.4. Thereafter tissues were embedded in paraffin and sections were cut for standard hematoxylin-and-eosin stain (Sigma). For immunohistochemical detection, antigen epitopes on tissue sections were retrieved by microwaving with 10 mM sodium citrate at pH 6.0, followed by quenching of endogenous peroxidase activity by 3% hydrogen peroxide, and blocking by 3% BSA. Sections were incubated with antibodies against human mitochondria (Millipore) with mouse IgG isotype as negative control. Color development with diaminobenzidine was performed by using Histostain Plus Kit according to the manufacturer's instructions (Invitrogen).

### Luciferase reporter assay

The human Hif-2α promoter DNA containing the PPAR-γ binding region with 5′ and 3′ flanking sequences was amplified by a pair of primers (F: 5′-**GAGAGCTAGC**TCTGGTACCTGCATGCCATA -3′, R: 5′-**CTCTAAGCT**TGTCATTGTTCCTGGCTGACC-3′), and subcloned between *NheI* and *HindIII* restriction sites (bold letters) immediately upstream of luciferase gene in the pGL3-basic vector (Promega). Point mutations (underlined letters) of Hif-2α binding sequence were obtained by PCR amplification of Hif-2α-pGL3 constructs with a primer pair (F: 5′-CCCTTAGGAACGAAGGACACATGAACCCAGATGCTGTTGCTC-3′, R: 5′-GAGCAACAGCATCTGGGTTCATGTGTCCTTCGTTCCTAAGGG-3′) by using Site-directed mutagenesis Kit (Promega). HUCPVCs were seeded in 24-well plates for 24 h, 900 ng PPAR-γ-expression vector (Origene) was co-transfected with 300 ng wild-type or mutated Hif-2α-pGL3 constructs for 24 h. 0.05 µg pRL-CMV plasmid expressing Renilla luciferase was co-transfected in each well to monitor the transfection efficiency (Promega). At 24 h post-transfection, firefly luciferase activity was measured by using dual-luciferase reporter assay as described by the manufacturer (Promega). Relative luciferase activity was normalized with Renilla luciferase activity in each well and then compared with the luciferase value of the empty pGL3-basic vector.

### Statistical analysis

For the quantitative real-time PCR, cell proliferation and colony formation assays, all the experiments were repeated at least three times unless otherwise stated. Comparisons were made by using student's *t* test. Results were expressed as mean ± standard deviation (SD). Significance level used was *P*<0.05.

## Results

### Isolation and purification of CD146^+^ HUCPVCs

**Figure 1:**
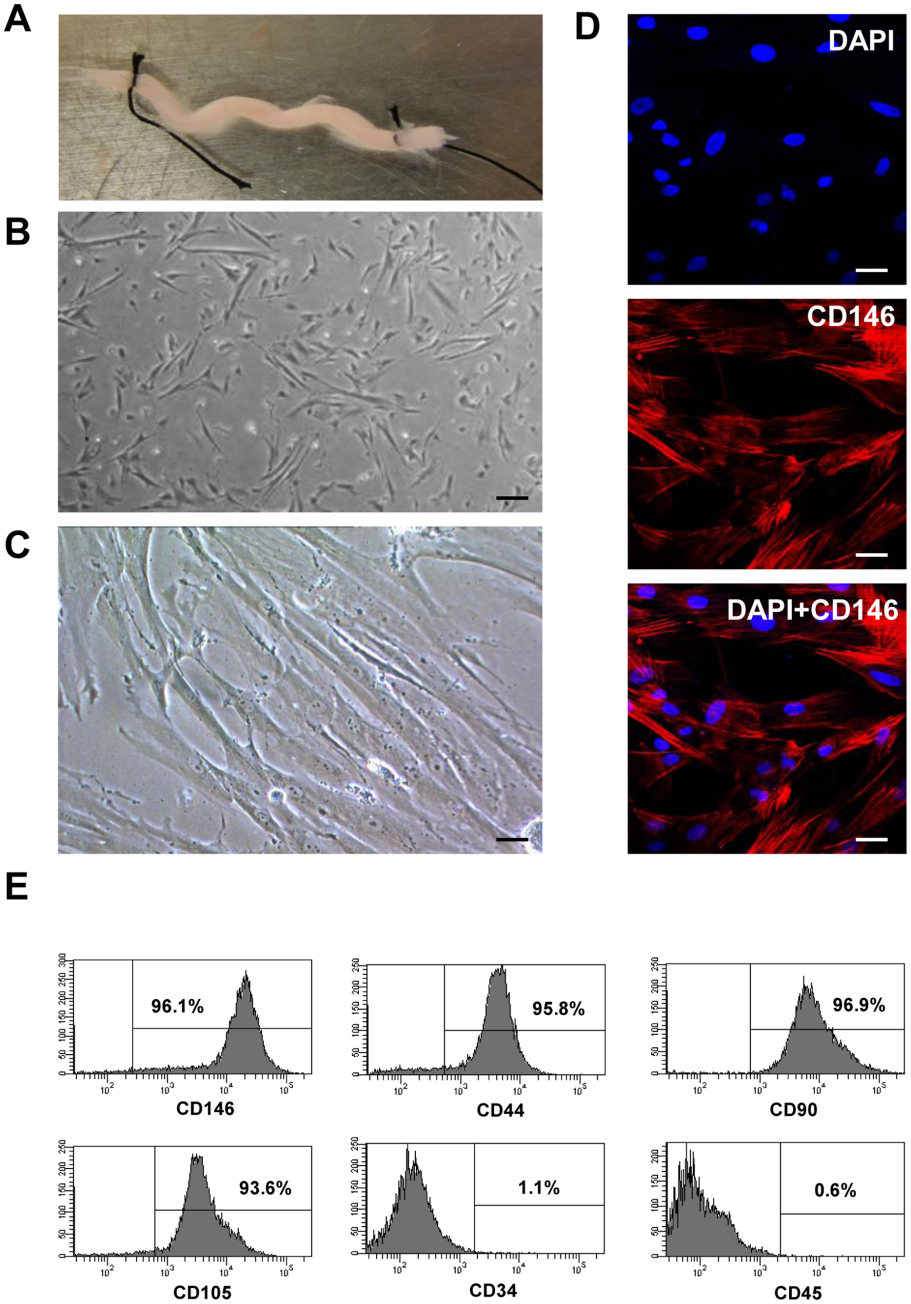
Isolation and purification of CD146^+^ HUCPVCs from human umbilical cord. A. A dissected human umbilical cord vessel. B. Heterogenous population of HUCPVCs digested from the umbilical cord vessel by collagenase I. Scale bar: 100 µm. C. Homogenous population of HUCPVCs purified with CD146^+^ antibody using MACS system. Scale bar: 50 µm. D. Expression of CD146 in HUCPVCs. Cells were immunostained with anti-CD146 antibody and visualized by fluorescent microscopy. The nucleus was counterstained with DAPI. Scale bar: 50 µm. E. Detection of cell surface markers by flow cytometry analysis. Cells were immunostained with selected cell surface markers CD146, CD44, CD90, CD105, CD34, CD45. The percentage of cell population with positive staining was shown in each figure.

We have extracted, isolated and purified CD146^+^ HUCPVCs cells from the perivascular region (i.e. the adventitial surface of the umbilical cord vessels) of human umbilical cords. After carefully cutting open the umbilical cord, the umbilical vessels (the vein and the two arteries) were taken and ligated at the two ends (Figure 1A) to prevent cross contamination with endothelial cells and cord blood hematopoietic stem cells. After digestion with collagenase I, a heterogeneous population of cells was collected (Figure 1B). The HUCPVCs were then purified by CD146-conjugated magnetic beads. By positively selecting cells expressing CD146, a surface antigen presenting in HUCPVCs, we achieved a more homogeneous HUCPVCs cell population which displayed a fibroblastic-like morphology (Figure 1C). Immunofluorescence staining of the isolated cells further confirmed a high level of CD146 expression in HUCPVCs (Figure 1D). Flow cytometry analysis of cells staining with CD146 antibody indicated that on the average 96.1% of cells was CD146 positive (Figure 1E). Of note, CD44, CD90 and CD105 surface markers were also highly expressed while the hematopoietic markers CD34 and CD45 were negative in CD146^+^ HUCPVCs.

### Multi-lineage differentiation potential of CD146^+^ HUCPVCs

To access the multilineage differentiation capacity, the differentiation potential of purified CD146^+^ HUCPVCs was accessed by culturing under defined differentiation conditions.

**Figure 2:**
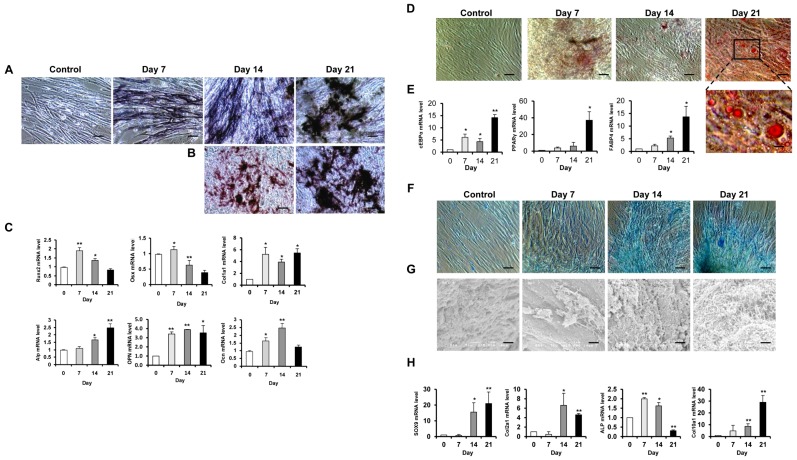
Multilineage differentiation of CD146^+^ HUCPVCs. Osteogenic differentiated CD146^+^ HUCPVCs at indicated days were fixed and processed for alkaline phosphatase (A) and Alizarin red S (B) staining. Cell morphology was captured by phase contrast microscopy. Scale bar: 100 µm. (C) Relative mRNA expression of osteogenic marker genes including Runx2, Alp, Ocn, Osx, Opn and Col1α1 was detected by quantitative real-time PCR. (D) For induction of adipogenic differentiation, the cells were incubated in adipogenic medium for indicated days and stained with Oil red O. Scale bar: 100 µm. (E) mRNA expression of adipogenic marker genes Cebpα, Ppar-γ and Fabp4 was examined by real-time PCR. (F) For chondrogenic differentiation, the cells were incubated in chondrogenic medium for indicated days and stained with Alican blue. Cell morphology was captured by phase contrast microscopy. Scale bar: 100 µm. (G) The presence of collagen fibers at day 21 of incubation was detected by scanning electron microscope Scale bar: 1 µm. (H) mRNA expression of chondrogenic marker genes Sox-9, Col2α1, Alp and Col10α1 by quantitative real-time PCR. Control: Cells without treatment. Values shown are mean ± SD (n = 3). *P<0.05; **P<0.01.

- **Osteogenic differentiation.** CD146^+^ HUCPVCs were induced first with osteogenic inducing medium for up to 21 days. Samples were collected on days 7, 14 and 21. Positive staining of alkaline phosphatase (ALP) in the extracellular matrix of HUCPVCs was observed at day 7 of osteogenic induction and the staining was remarkably increased at days 14 and 21 (Figure 2A). Consistent with the ALP activity result, extracellular matrix mineralization was observed upon osteogenic induction at day 14 and to be further increased at day 21 (Figure 2B). In addition, the mRNA expression of osteogenic marker genes Alp, Ocn, Opn and Col1α1 was upregulated following osteogenic induction (Figure 2C). Of note, Runx2 was mostly upregulated at day 7 and then decreased at days 14 and 21.
- **Adipogenic differentiation.** Upon adipogenic induction, the CD146^+^ HUCPVCs gradually accumulated the Oil Red O positive lipids (Figure 2D), with a steady elevation of the transcription of adipogenic markers Cebpα, Ppar-γ and Fabp4 (Figure 2E). The expression of these genes was in lined with the increase in size of the lipid droplets from day 7 to day 21.
- **Chondrogenic differentiation.** CD146^+^ HUCPVCs showed a high content of cartilage specific proteoglycans in the cell mass culture (Figure 2F) after 14 days of chondrogenic induction and this was more evident at day 21. The presence of collagen fibers or abundant collagen synthesis, which was one of the characteristics for chondrocytes, was also observed via the scanning electron micrographs at day 21(Figure 2G), The mRNA expression of Sox9, Col2α1, Col10α1 kept increasing until day 21 following the induction. Alp mRNA was also increased at days 7 and 14 while decreased expression was observed at day 21, the maturation stage of chondrogenic differentiation (Figure 2H).

### CD146^+^ HUCPVCs as a cell source for bone regeneration

**Figure 3:**
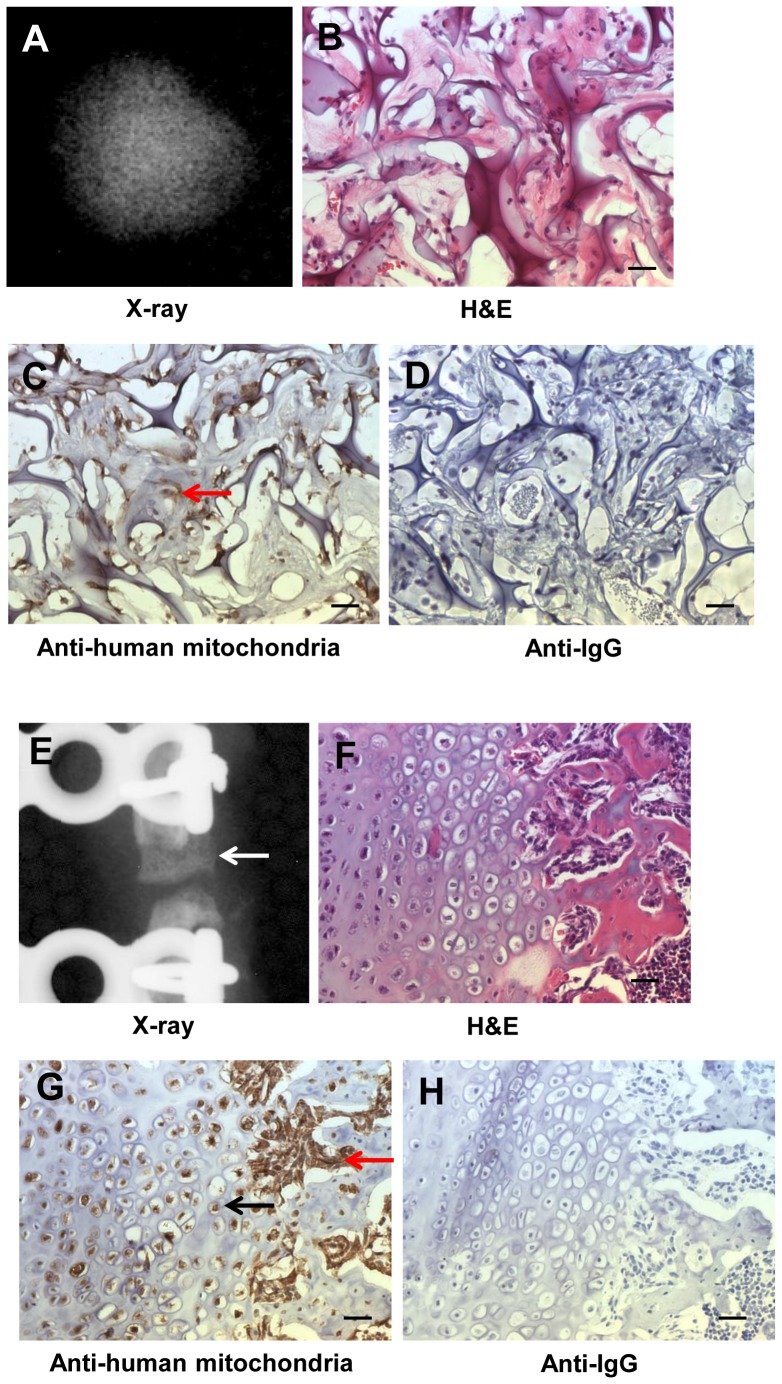
Functional engraftment of CD146^+^ HUCPVCs in the new bone regenerates in the subcutaneous transplantation model and the critical-sized bone defect model in SCID mice. (**A**) X-ray image of the ectopic bone formation at 6 weeks following CD146^+^ HUCPVCs transplantation subcutaneously in the back of SCID mice (n = 3). (**B**) H&E staining of the ectopic bone section. (**C**) Immunohistochemistry staining for human specific mitochondria in the newly formed ectopic bone (**D**) Mouse IgG isotype control. (**E**) X-ray image of critical sized bone defect region at 6 weeks following CD146^+^ HUCPVCs transplantation. The newly formed bone is indicated by the white arrow. (**F**) H&E staining of the new bone regenerate. (**G**) Immunohistochemistry staining for human specific mitochondria in the new bone regenerate in the critical-sized bone defect model. (**H**) Mouse IgG isotype control. Red arrow: Osteoblasts; Black arrow: Chondrocytes. All scale bar: 50 µm.

To examine whether CD146^+^ HUCPVCs can participate in the *in vivo* osteogenesis, we performed an ectopic bone formation model where CD146^+^ HUCPVCs were seeded in a 3D Gelfoam-alginate complex and were transplanted subcutaneously in the back of the SCID mice. After 6 weeks, X-ray images showed ectopic mineralization of the transplants (Figure 3A). Histological staining of the sections of the transplants revealed newly formed bony matrix with osteocytes embedded in the matrix (Figure 3B). Human specific mitochondria immunostaining suggested that the osteocytes embedded in the bone matrix were donor origin (Figure 3C, D). To further determine the functional engraftment of CD146^+^ HUCPVCs in the new bone regenerates during bone repair or regeneration, we employed a critical-sized bone defect model in the SCID mice. Similar with the subcutaneous bone formation model, X-ray examination showed newly formed bone in the defect region at 6 weeks following CD146^+^ HUCPVCs transplantation (Figure 3E). Both cartilaginous callus and bony callus formed in the regenerated new bone (Figure 3F). Positive staining for human specific mitochondria suggested that the CD146^+^ HUCPVCs were functionally engrafted in the new bone regenerate to involve in the regeneration of the critical-sized bone defect (Figure 3G, H).

### Hypoxia inhibits the osteogenic differentiation of CD146^+^ HUCPVCs while sustains their multi-differentiation capability

**Figure 4:**
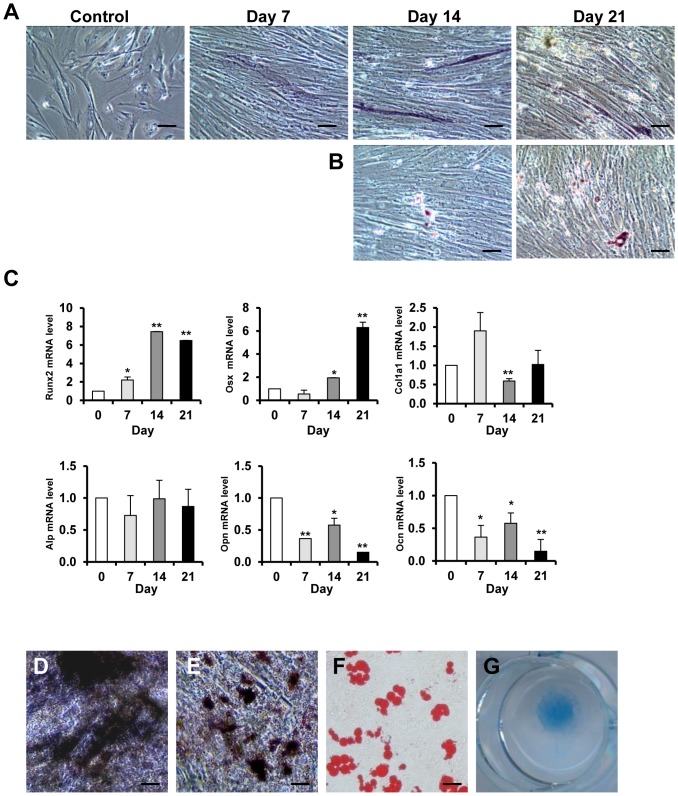
Hypoxia inhibits osteogenic differentiation of CD146^+^ HUCPVCs while maintains their multi-differentiation potential. Osteogenic differentiated CD146^+^ HUCPVCs at indicated days were fixed and subject to alkaline phosphatase (A) and Alizarin red S (B) staining respectively. Control: Cells without treatment. (C) Relative mRNA expression of osteogenic marker genes including Runx2, Alp, Ocn, Osx, Opn and Col1a1 was detected by quantitative real-time PCR. Multilineage induction of hypoxia expanded CD146^+^ HUCPVCs was performed using defined conditions as described in Materials and Methods part, and the cells were processed for cytochemistry staining with (D) alkaline phosphatase, (E) Alizarin Red S, (F) Oil Red O, and (G) Alican Blue. Values shown are mean ± SD (n = 3). *P<0.05; **P<0.01. Scale bar: 100 µm.

We next examine the effect of low oxygen tension on osteogenic differentiation in CD146^+^ HUCPVCs. Cells were cultured in osteogenic medium at 2% oxygen concentration (hypoxia) for 7, 14 and 21 days. There was no remarkably increase of alkaline phosophatse in the cultures when compared to the no induction control (Figure 4A). Mineralized matrix formation in the cultures at 14 and 21 days of osteogenic induction in hypoxic condition was much less than that under normoxia (Figure 4B and Figure 2B). In concord, the osteogenic related genes in CD146^+^ HUCPVCs under hypoxia displayed a different expression pattern from that in normoxic conditions, with no significant change in Alp mRNA expression, an upregulation in Runx2 and Osx but a downregulation of Col1α1, Opn and Ocn during the 21 days of induction (Figure 4C). These results suggested that low oxygen microenvironment inhibited osteogenic differentiation of CD146^+^ HUCPVCs. We next asked whether the suppressed osteogenesis is due to a loss of differentiation ability in CD146^+^ HUCPVCs or a more sustainable primitive state induced by the low oxygen tension. To address this question, we cultured the CD146^+^ HUCPVCs at 2% oxygen for two passages (14 Days) and restored the cells to normoxic conditions. The cells were then subjected to osteogenic, adipogenic, and chondrogenic differentiation under defined induction conditions as described above. After 14 days of induction, strongly positive staining of ALP (Figure 4D) and Alizarin red S (Figure 4E) were observed, indicating a robust osteogenic differentiation. Adipogenic and chondrogenic differentiation ability of the cells were also evident by the accumulation of lipid droplets (Figure 4F) and the proteoglycan synthesis (Figure 4G), respectively. These data implied that hypoxia maintained CD146^+^ HUCPVCs at an early progenitor stage and sustained their multi-lineage differentiation capability.

### Hypoxia promotes the colony forming efficiency and proliferation of CD146^+^ HUCPVCs

**Figure 5:**
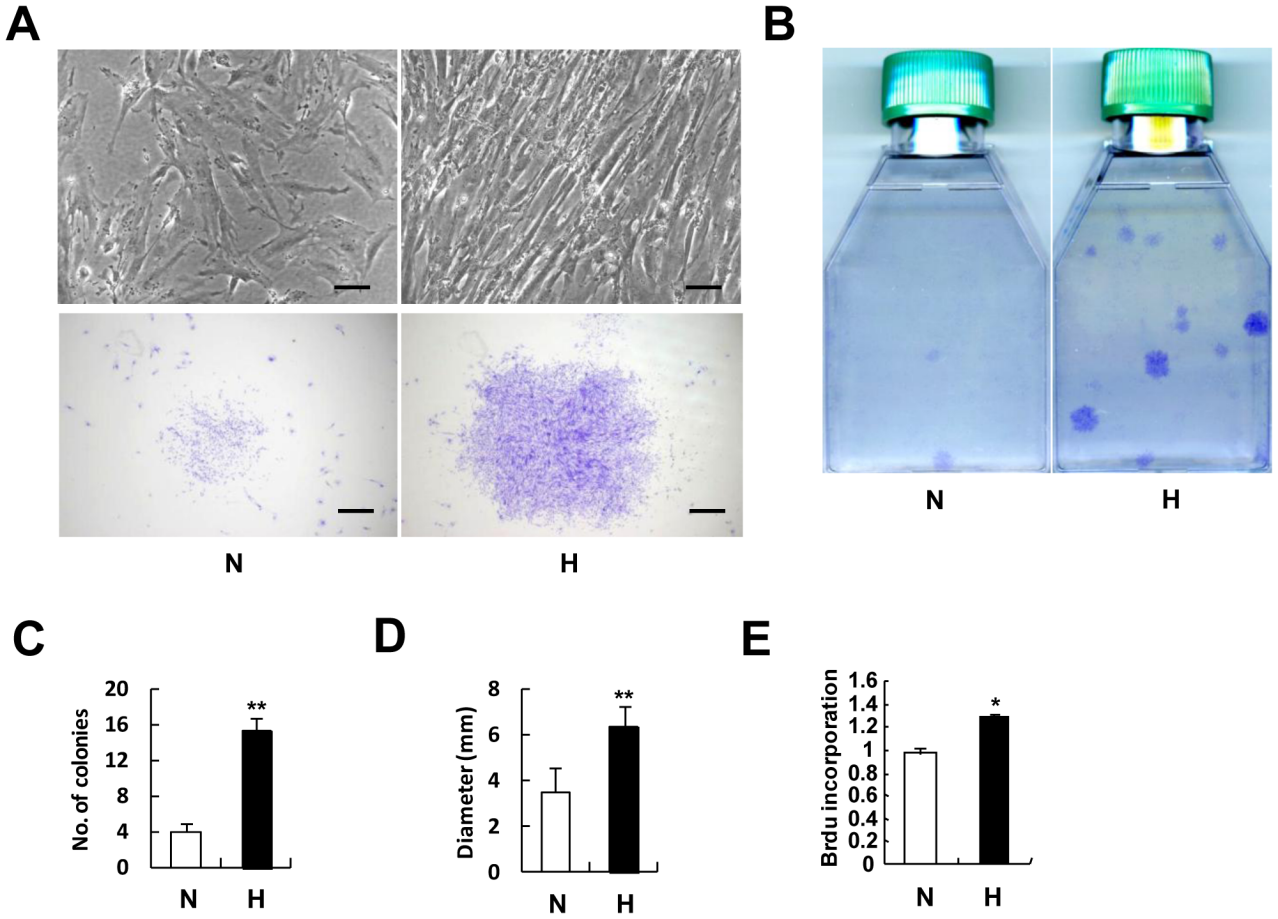
Hypoxia increases colony forming efficiency and proliferation of CD146^+^ HUCPVCs. (A) Cell morphology captured by phase contrast microscopy (upper) and the representative colonies for each treatment were shown (lower). The colonies in CD146^+^ HUCPVCs culture under normoxic or hypoxic conditions were visualized by cells staining with crystal violet (B). The total numbers of colonies as well as the average diameters of colonies was quantified in (C) and (D) respectively. (E) BrdU incorporation in CD146^+^ HUCPVCs after 48 hours of normoxia or hypoxia exposure. N: Normoxia; H: Hypoxia. Values shown are mean ± SD (n = 3). *P<0.05; **P<0.01. Scale bar: 100 µm.

To further confirm whether hypoxia maintains CD146^+^ HUCPVCs at an undifferentiated state, we measured their colony forming efficiency and performed the BrdU incorporation assays. As shown in Figure 5, low oxygen condition increased the clonogenic potential of CD146^+^ HUCPVCs. Over the same culture time, cells cultured in hypoxia gave rise to densely packed large colonies while cells cultured at the same density under normoxic condition only formed loosely packaged small colonies (Figure 5A) . The numbers of colony forming units per flask increased by more than 3 fold in hypoxia cultures as compared to that in normoxia (Figure 5B and 5C). The average size of individual colonies under hypoxia was approximately two times bigger than that under normoxia (Figure 5D). After pulse labeling cells with BrdU for 4 hours, CD146^+^ HUCPVCs exposed to hypoxia for 2 days contained 30% more S-phase cells (cells incorporated with BrdU) than those under normoxia (Figure 5E), implying that hypoxia moved more cells into an active cell cycle. Taken together, our data suggested that low oxygen microenvironment provided a self-renewal-promoting niche which not only effectively maintained CD146^+^ HUCPVCs' stemness but also enhanced its clonogenicity and proliferation *in vitro*.

### Hypoxia significantly alters the expression profile of the stem cell transcriptional regulatory network in CD146^+^ HUCPVCs

**Figure 6:**
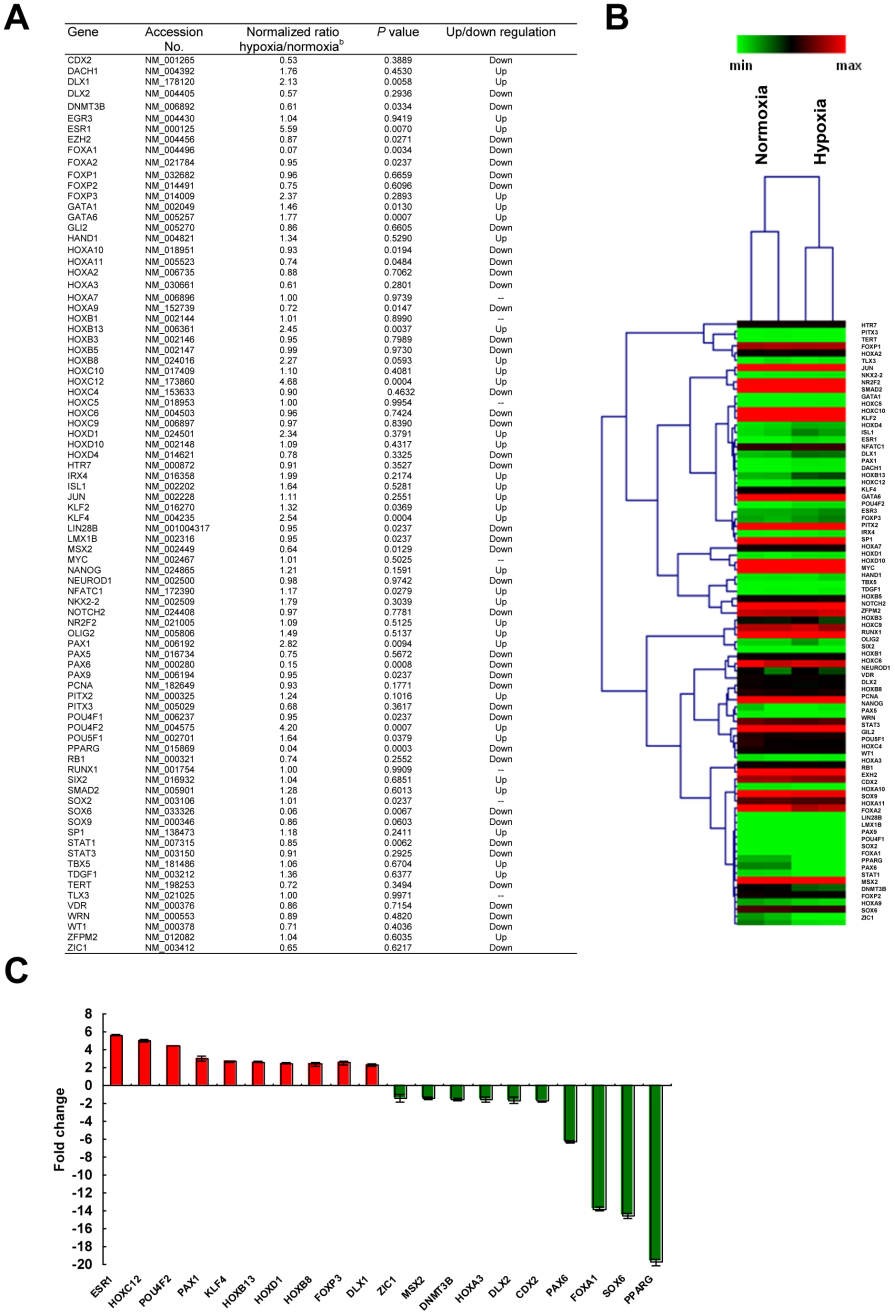
Expression profiles of stem cell transcription factors in CD146^+^ HUCPVCs under hypoxia and normoxia. (A) The table summarizes the fold-change of mRNA expression of stem cell transcription factors in CD146^+^ HUCPVCs in response to hypoxia. (B) Hierarchical clustering analysis on the PCR array data. (C) Illustration of the mRNA fold-change of the top ten upregulated and downregulated transcription factors in CD146^+^ HUCPVCs in response to hypoxia.

To better understand the molecular basis of the regulation of self-renewal by hypoxia, and to identify the stem cell related transcription factors that are selectively activated or repressed in response to hypoxia, a pathway-focused PCR array was performed in HUCPVCs. The gene expression profile of 84 stem cell transcription factors in CD146^+^ HUCPVCs subjected to normoxia and hypoxia exposure was shown in Figure 6A and B. 34 genes were found to be up-regulated in response to hypoxia, particularly Esr1 (∼6-fold), Hoxc12 (∼5-fold) and Pou4F2 (4.5-fold). On the other hand, 43 genes were detected as down-regulated. Ppar-γ was the mostly suppressed gene under hypoxic conditions (∼20-fold), followed by Sox6 (∼15-fold) and Foxa1 (∼14-fold) (Figure 6C). There was no significant change in the expression of 7 genes including Hoxa7, Hoxb1, Hoxc5, Myc, Runx1, Sox2 and Vdr. These data clearly demonstrated that hypoxia significantly changed many key genes associated with stem cell maintenance, differentiation and development in the transcriptional regulatory network in CD146^+^ HUCPVCs.

### Hypoxia inhibits transcriptional suppression of HIF-2α by PPAR-γ in CD146^+^ HUCPVCs

**Figure 7:**
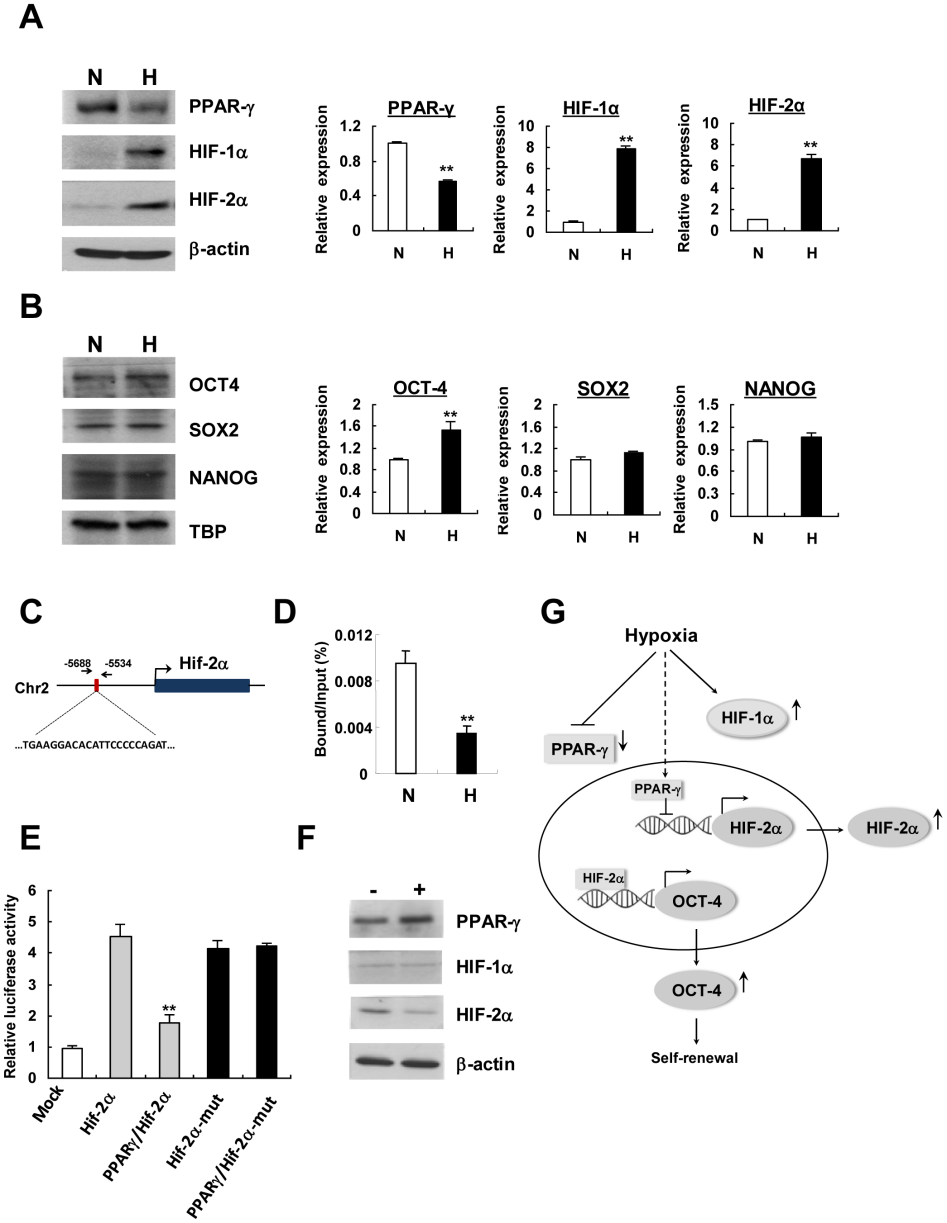
Hypoxia inhibits transcriptional suppression of Hif-2α by PPAR-γ in CD146^+^ HUCPVCs. Protein expression of PPAR-γ and HIFs (A) as well as pluripotency marker proteins (B) was examined by Western blot analysis. Relative protein expression was quantified by densitometry. The results shown are representative of three independent experiments. (C) Schematic outline of Hif-2α DNA with promoter region harboring the putative binding site of PPAR-γ. Nucleotide sequence for putative binding was shown. The position of the region amplified by PCR after chromatin immunoprecipitation was shown by arrows. (D) ChIP assay. Cells were exposed to normoxic or hypoxic conditions for 24 h before chromatin DNA extraction. The percentage of DNA bound was calculated by dividing the immunoprecipitated DNA with the total amount of DNA purified from the chromatin (input DNA). N: Normoxia; H: Hypoxia. (E) Luciferase activity of HUCPVCs after co-transfection of PPAR-γ expression vector with Hif-2α-pGL3 wild-type or mutated reporter construct. Luciferase activity was measured by dual-luciferase reporter assay (Promega) and was normalized to Renilla luciferase activity. Relative luciferase activity of each sample was compared to that transfected with empty pGL3-basic vector (mock). Values shown are mean ± SD (n = 3). **P<0.01. (F) Western blot analysis of HIF-1α and HIF-2α protein expression upon PPAR-γ overexpression. Cells were transfected with an empty vector (−) or PPAR-γ-expression vector (+) before protein extraction. (G) A suggested model of PPAR-γ and HIF-2α expression in regulation of self-renewal of CD146^+^ HUCPVCs in response to hypoxia.

We next confirmed the protein expression of PPAR-γ in response to hypoxia. Western blot analysis showed a 50% decrease of PPAR-γ protein expression in CD146^+^ HUCPVCs after hypoxia exposure (Figure 7A). The result was in line with its mRNA suppression from the PCR array data (Figure 6). As expected, low oxygen environment strongly induced HIF-1α (7.8-fold) and HIF-2α (6.7-fold) expression in CD146^+^ HUCPVCs (Figure 7A). To examine whether an enhancement of cell clonogenicity and proliferation by hypoxia was related to changes in expression of pluripotency marker genes, we measured the protein levels of OCT4 (POU5F1), SOX2 and NANOG by Western blot analysis. About 1.5-fold increase of OCT4 expression was observed in hypoxic condition while there was no significant change in SOX2 and NANOG expression (Figure 7B). The result was coincided with their mRNA expression obtained from PCR array analysis (Figure 6).

As a transcription factor exerts its function by binding to specific sequences of DNA adjacent to the genes that it regulates, we then searched the PPAR-γ consensus binding site in the promoter regions of Hif-1α and Hif-2α genes. A putative PPAR-γ consensus binding site located 5.6 kb upstream of transcriptional start site of the Hif-2α gene was identified (Figure 7C) while no consensus binding site was found in the promoter region of Hif-1α gene. To verify the binding of PPAR-γ to the Hif-2α promoter, chromatin immunoprecipitation assay was performed. CD146^+^ HUCPVCs were exposed to either normoxic or hypoxic conditions for 24 h. Cross-linked chromatin was then immunoprecipitated with antibodies against PPAR-γ or no-antibody hybridization as negative control. Quantitative PCR was performed on the immunprecipitated DNA and on total input DNA (input) by using primers flanking the putative binding sequences. As shown in Figure 7D, Hif-2α DNA bound with PPAR-γ was readily detected in cells cultured under normoxia but dramatically decreased when the cells grew under hypoxia, demonstrating that PPAR-γ binds to Hif-2α promoter region and the binding was suppressed in response to hypoxia. To further confirm whether the binding of PPAR-γ may lead to transcriptional control of Hif-2α gene, luciferase reporter assays were performed by co-transfection of PPAR-γ-expressing vector with a luciferase reporter construct harboring the Hif-2α promoter binding site. Results indicated that the luciferase activity of Hif-2α gene promoter was suppressed by co-transfection with PPAR-γ-expressing constructs, but the repression was attenuated when the binding site of Hif-2α gene promoter was mutated (Figure 7E). This indicates that PPAR-γ not only binds to Hif-2α gene promoter but also acts as a transcriptional repressor of Hif-2α expression. PPAR-γ overexpression led to downregulation of HIF-2α protein expression but there was no apparent change of HIF-1α expression in CD146^+^ HUCPVCs (Figure 7F). This finding echoed to the coordinated suppression of PPAR-γ expression with an activation of HIF-2α (Figure 7A) and the upregulation of Oct4 mRNA (Figure 6A) and protein (Figure 7B) expression, a known down-stream gene regulated by HIF-2α. Therefore, PPAR-γ may participate in HIF-2α dependent regulation of Oct-4 in stem cell self-renewal (Figure 7G).

## Discussion

The bone marrow derived MSCs are the most extensively characterized type of mesoderm origin adult stem cells and have been applied for skeletal tissue repair and regeneration [2], [3], [23], [24]. It is recently shown that MSCs can be isolated as perivascular cells in multiple human tissues including bone marrow, connective tissues and solid organs [24]. Among those alternative sources of mesenchymal progenitors, HUCPVCs holds particular interest for regenerative medicine community. HUCPVCs are rich in source, highly clonogenic [14], biologically potent [24], immunosuppressive and non-alloreactive [25]. Therefore they are a promising candidate for regeneration therapy. However, it has been challenging to obtain sufficient amount of HUCPVCs numbers in a given period of time for clinical application. Molecular and cellular mechanisms that control the self-renewal and differentiation of HUCPVCs remain to be elucidated. Moreover, evidence is insufficient whether this population can functionally engraft during tissue repair and regeneration.

HUCPVCs have been characterized to express cell surface antigens, including CD44, CD73, CD90, CD105, CD106, CD146 and 3G5 [24]–[26]. In this study, we combined traditional adherent culture with the magnetic cell sorting method to enrich HUCPVCs from the heterogenous early-passage primary cultures by using CD146-conjugated magnetic beads. The purified CD146^+^ HUCPVCs were morphologically homogeneous (Figure 1C) and also phenotypically homogeneous (Figure 1E) in the strong expression of putative human MSCs markers CD44, CD90 and CD105, and lacking of the hematopoietic markers CD34 and CD45, suggesting that CD146-based immunomagnetic purification could serve as a time-efficient approach to obtain enriched phenotype-defined HUCPVCs population from bulk culture.

Purified CD146^+^ HUCPVCs were highly proliferative and potent in mesenchymal lineage differentiation both *in vitro* and *in vivo* (Figure 2 and 3A–3D). Thus it is also an enriched functional defined MSC population. Following transplantation into the critical-sized bone defect sites in SCID mice, CD146^+^ HUCPVCs actively participate in the new bone regeneration process, forming cartilaginous callus and bony callus (Figure 3E–3H). Our data provided the evidence that CD146^+^ HUCPVCs functionally engrafted in skeletal tissue regeneration and might serve as a promising cell source for cell based therapy.

Adult bone marrow MSCs have been documented to display limited *in vitro* self-renewal [27], [28]. After a certain number of cell divisions, bone marrow MSCs enter senescence which is characterized by enlarged and irregular cell shapes, with reduced differentiation potential [27], [28]. Although HUCPVCs have been shown to maintain a high clonogenic frequency *in vitro* [14], aging could still be a potential problem for HUCPVCs in prolonged culture. Excitingly, exposure of CD146^+^ HUCPVCs to low oxygen tension dramatically promoted its expansion and significantly increased the colony forming efficiency compared with the cultures under normoxia (Figure 5). The extensive cell divisions were accompanied by suppressed osteoblastic differentiation (Figure 4A) and were also associated with a state that the mesenchymal lineage differentiation capacity was fully sustained. When restored to the favorable differentiation promoting environment (normoxia plus specific lineage differentiation conditions), CD146^+^ HUCPVCs were readily differentiated into osteoblasts, adipocytes and chondrocytes (Figure 4D–4G). It is therefore suggested that hypoxia may provide CD146^+^ HUCPVCs a microenvironment (or niche) in favor of self-renewing division.

HIFs are transcription factors that play a central role in cellular responses to changes in tissue oxygen concentrations. The α subunit of HIFs is stabilized under hypoxic condition and translocates from cytoplasm into the nucleus where it dimerizes with HIF-1β to transactivate downstream target genes. Bone loss induced by hypoxia occurs at pathological conditions such as ischemia and vascular diseases. Hypoxia has been shown to inhibit osteogenesis in human MSCs through direct downregulation of RUNX2 by TWIST [29]. Twist, a basic helix-loop-helix transcription factor, is known as one of the downstream targets of HIF-1α. These may explain the suppressed osteoblastic differentiation of CD146^+^ HUCPVCs under hypoxia in our study.

Low oxygen microenvironment induced the expression of both HIF-1α and HIF-2α in CD146^+^ HUCPVCs (Figure 7A). Despite Hif-2α shares 48% sequence identity with Hif-1α and are structurally similar in their DNA binding, they act on overlapping but distinct target genes [30]. It has been shown that HIF-2α but not HIF-1α is direct upstream regulator of Oct-4, a well known stem cell pluripotency marker [17], [18]. Besides Oct-4, Sox2 and Nanog were also shown to be modulated by HIF-2α [18]. Both Oct-4 protein and mRNA were found to be upregulated by hypoxia in CD146^+^ HUCPVCs, but there was no remarkable change in protein levels of SOX2 and NANOG in the present study (Figure 7B). Hypoxia-enhanced self-renewal expansion in CD146^+^ HUCPVCs prompted us to establish the functional relationship between HIF-2α and key ‘stemness’ or self-renewal regulators. Through a stem cell transcription factor profiling by PCR array in CD146^+^ HUCPVCs, we identified Ppar-γ was markedly suppressed (Figure 6) by hypoxia, which was in agreement with the reduced protein level in hypoxic cells (Figure 7A). PPARγ plays a critical role in transactivation of most adipocyte-specific genes during adipocyte differentiation as well as insulin-responsive glucose uptake in adipocytes [31], [32]. Results from ChIP assay indicated a direct binding of PPAR-γ to the Hif-2α promoter *in vivo* and the binding was remarkably reduced in hypoxia (Figure 7D). In fact, PPAR-γ inhibition by hypoxia has been shown to be regulated by HIF-1 regulated gene DEC1/Stra13 during adipocyte differentiation in mouse embryonic fibroblasts [33]. Our data suggested that PPAR-γ might function as a repressor for Hif-2α transcription by binding to its promoter. This was supported by the suppression of luciferase activity of Hif-2α gene promoter by co-transfection with PPAR-γ expression construct. Transcriptional suppression of Hif-2α promoter by PPAR-γ expression did not occur when the binding sequence of the promoter was mutated (Figure 7E). Under hypoxia, the reduced binding of PPAR-γ to the Hif-2α promoter region resulted in an increased accumulation of HIF-2α followed by the upregulation of downstream targets of Hif-2α, such as Oct-4, that might control the self-renewal of CD146^+^ HUCPVCs (Figure 7G).

The expression of many other transcription factors associated with stem cell maintenance, differentiation and development were also changed in CD146^+^ HUCPVCs in response to hypoxia. What exact regulatory functions of those genes in controlling the self-renewal and differentiation of CD146^+^ HUCPVCs await further investigations.
